# Restored CD8^+^PD-1^+^ T Cells Facilitate the Response to Anti-PD-1 for Patients With Pancreatic Ductal Adenocarcinoma

**DOI:** 10.3389/fonc.2022.837560

**Published:** 2022-04-11

**Authors:** Qian Zhu, Guoliang Qiao, Lefu Huang, Chang Xu, Deliang Guo, Shuo Wang, Jing Zhao, Yuguang Song, Bing Liu, Zheng Chen, Zhiyong Yang, Yufeng Yuan

**Affiliations:** ^1^ Department of Hepatobiliary and Pancreatic Surgery, Zhongnan Hospital of Wuhan University, Wuhan, China; ^2^ Department of Surgical Oncology, Massachusetts General Hospital, Boston, MA, United States; ^3^ Department of Medical Oncology, Beijing Key Laboratory for Therapeutic Cancer Vaccines, Capital Medical University Cancer Center, Beijing Shijitan Hospital, Capital Medical University, Beijing, China; ^4^ First Department of Biliary Surgery, Eastern Hepatobiliary Surgery Hospital, Naval Military Medical University, Shanghai, China; ^5^ Department of General Surgery, Chinese People’s Liberation Army General Hospital, Beijing, China; ^6^ Department of Dermatology, The Central Hospital of Wuhan, Tongji Medical College, Huazhong University of Science and Technology, Wuhan, China; ^7^ Department of General Surgery, Huo Jianjun General Hospital, Beijing, China; ^8^ Department of General Surgery, Capital Institute of Pediatrics, Beijing, China

**Keywords:** CD8^+^PD-1^+^ T cell, TCR, PDAC, tumor resection, adoptive cell immunotherapy

## Abstract

**Purpose:**

We aimed to investigate the restoration of CD8^+^PD-1^+^ T cells through adoptive T-cell therapy (ACT) in relation to the prognosis and the therapeutic response to anti-PD-1 in patients with advanced pancreatic cancer (APC).

**Methods:**

A total of 177 adult patients who underwent tumor resection as initial treatment for pancreatic ductal adenocarcinoma (PDAC) from February 2013 to July 2019 at Zhongnan Hospital of Wuhan University were enrolled in this study. Another cohort of 32 patients with APC was prospectively enrolled from Capital Medical University Cancer Center between June 1, 2013, and May 30, 2019.

**Results:**

Of the 177 patients who received tumor resection, 67 tumor samples showed overexpression of PD-L1 and 110 patients with low expression of PD-L1. We found that overexpressed PD-L1 was a significant prognostic factor related to overall survival (OS). Furthermore, we tested the percentage of peripheral CD8^+^PD-1^+^ T cells in all patients and found that it was significantly correlated with the PD-L1 expression and the prognosis of patients with PDAC. The peripheral blood T lymphocyte subtypes were tracked for 30 months, and CD8^+^PD-1^+^ cells were shown to decrease. After that, we performed ACT for patients with APC in another cancer center. We found that the ratios of posttreatment of ACT/pre-ACT CD8^+^PD-1^+^ T cells were significantly related to the prognosis of patients with APC. Moreover, patients with combined treatment of ACT with anti-PD-1 had significantly favorable OS.

**Conclusions:**

This study showed that the CD8^+^PD-1^+^ T-cell level was related to the expression of PD-L1. Restoring CD8^+^PD-1^+^ T cells in patients with APC by treatment of ACT significantly benefits the prognosis and facilitates the response to anti-PD-1.

## Introduction

Pancreatic ductal adenocarcinoma (PDAC) is one of the most aggressive solid malignancies and the leading cause of cancer-related deaths. Surgical resection is the main radical treatment, but less than 20% of patients have a resectable tumor at the time of diagnosis (1, 2). Immunotherapy is among the most promising strategies under development but is complicated by the immunosuppressive tumor microenvironment prevalent in PDAC (3, 4). Programmed death receptor 1 (PD-1) is one of the most important checkpoint pathways and has been approved to be used in various cancers (5, 6). Several investigations have shown that anti-PD-1 is not effective in patients with PDAC since targeting this pathway should induce T-cell activity and consequently cancer cell death (7). However, the prognosis has not given satisfactory results in patients with advanced pancreatic cancer (APC) who received immunotherapy (8–10). Nevertheless, adoptive cell therapy (ACT), delivery of *ex vivo* activated cellular products such as dendritic cells (DCs), NK, and/or T cells, has shown activity in pancreatic cancer. Widespread use of ACT is enabled by practical generation processes, rapid expansion *ex vivo*, and MHC-unrestricted tumor cell killing. Further, it is feasible to combine ACT with chemotherapy as we previously demonstrated in patients with APC (11).

The ACT product is a complex mixture of cell types, and we hypothesize that greater efficacy would be achieved by a better understanding of which cell subtypes affect the clinical outcome so that they may be modulated (12). We have observed an increase in CD8^+^ T cells that express programmed death 1 (PD-1, CD279) within expanded cellular products. PD-1 expressed by CD4^+^ and CD8^+^ T cells is often viewed as a co-inhibitory receptor and induced by T-cell receptor (TCR) signaling, the engagement of which by its ligands PD-L1 and PD-L2 on tumors and other immune cells impairs effector T-cell function (13). Previous studies showed that PD-1 expression levels in virus-specific peripheral blood CD8^+^ T cells correlated with the disease progression of some viral infections in humans (14–16). Tumor-infiltrating lymphocytes (TILs) that express high levels of PD-1 are functionally impaired, failing to produce cytokines such as interleukin 2 (17–20), and the presence of intratumorally PD-1 signaling has been associated with worse survival in PDAC (21). However, PD-1 is also a marker of activated T cells and may identify a population of T cells with potential antitumor activity. Rosenberg *et al.* observed that CD8^+^PD-1^+^ TILs recovered reactivity after exposure to high-dose IL-2, resulting in higher tumor-specific IFN-γ production compared with CD8^+^PD1^−^ T cells (20).

Therefore, in the present study, we hypothesized that CD8^+^PD-1^+^ T cells were the cell subset that gradually exhausted after tumor resection in selected patients, and restoring the amount of CD8^+^PD-1^+^ T cells through ACT could improve the prognosis and facilitate the therapeutic response to anti-PD-1 in patients with APC.

## Patients and Methods

### Source of Patients and Clinical Specimens

A total of 177 adult patients (age > 18 years) who underwent tumor resection as initial treatment for PDAC from February 2013 to July 2019 at Zhongnan Hospital of Wuhan University were enrolled in this study. The inclusion criteria were as follows: 1) no anticancer treatments before enrollment; 2) no additional adjuvant chemotherapy routinely administered unless a recurrence was identified; and 3) no other malignancies simultaneously. The exclusion criteria were as follows: 1) repeat tumor resection and 2) presence of cardiac, pulmonary, or renal insufficiency before operation. The patients’ clinical data were retrospectively collected and included demographics, body mass index (BMI), and preoperative CA19-9 levels. Postoperative outcomes and treatment included the occurrence of major morbidity (Clavien–Dindo ≥ III) and 30-day mortality. Pathological parameters were collected according to the 8th edition of the American Joint Committee on Cancer (AJCC) TNM staging system and included tumor stage, tumor size, extent of lymph node involvement, and tumor grading. Phenotypic analysis of peripheral blood immune cells was tested and followed up every month after surgery, and other follow-up data were obtained from their most recent medical review, which consisted of a clinical examination and an assessment of CT scans. Patients’ overall survival (OS) time was calculated from the surgery date to the date of death or last contact. An independent biostatistician managed and maintained the collected data.

Another cohort of 32 patients with advanced PDAC was enrolled from Capital Medical University Cancer Center, Beijing Shijitan Hospital from June 1, 2013, to May 30, 2019. The study was approved by the Regional Ethical Review Board for Capital Medical University Cancer Center. Patients were treated according to the Declaration of Helsinki’s ethical principles for medical research involving human subjects. All patients provided informed written consent prior to study entry. All patients underwent ACT, and 15 of 32 (46.8%) patients received ACT combined with anti-PD-1 (pembrolizumab). Patients’ OS time was calculated from the ACT treatment date to the date of death or last contact.

### Preparation of Adoptive T-Cell Therapy Product for Sorting

The ACT product was generated *ex vivo* as described in detail previously (22). Peripheral blood stem cells were mobilized by injection of granulocyte-macrophage colony-stimulating factor (GM-CSF) measuring 5 μg/kg per day (Chugai Pharm Co. Ltd., Tokyo, Japan) until the level of mononuclear cells in peripheral blood reached 1.5 × 10^9^/L. Then, peripheral blood mononuclear cells (PBMCs) were collected by a COBE Spectra cell separator (COBE BCT, Lakewood, CO, USA) until the CD34^+^ count reached a threshold of 4.5 × 10^6^/kg. All collections were frozen at −80°C until required for further analysis. Between 30 and 50 ml of thawed apheresis product was co-cultured 7 days with IL-4 (1,000 U/ml; R&D Systems, Inc., Minneapolis, MN, USA), TNF-α (20 ng/ml; R&D Systems, Inc., Minneapolis, MN, USA), and GM-CSF (800 U/ml; Amoytop Biotech Co., Ltd., Xiamen, China) to generate autologous DCs. Another aliquot of PBMCs was expanded in a complete medium consisting of AIM-V supplemented with 10% heat-inactivated human AB serum and the recombinant cytokines IL-2 at 2,000 U/ml (Boehringer Mannheim, Mannheim, Germany) and CD3 antibody at 1.7 mg/ml (Boehringer Mannheim, Germany). Subsequently, half of the media was replaced with fresh AIM-V containing IL-2 (2,000 IU/ml) every other day. After 7–10 days, the autologous DCs were mixed with cultured cytotoxic T lymphocytes (CTLs) at a ratio of 1:100 for 7 days, and then the co-cultured DC–CTLs were harvested.

### Generation and Sorting of CD8^+^PD-1^+^ T Cells

The CD8^+^PD-1^+^ T cells were sorted from the *ex vivo* expanded T-cell products. Cell counts were recorded daily from day 0 to day 30. The sorted CD8^+^PD-1^+^ T cells were tested for antitumor activity assay *in vitro*. The TCR repertoire of cultured cells was performed at day 0 and day 30 to determine the association with peripheral blood lymphocyte phenotype after ACT infusion and subsequent clinical response.

### Flow Cytometric Analysis and Sorting

We used the following fluorochrome-conjugated antibodies: CD3 PerCP-Cy5.5, CD4 FITC, CD8 FITC, CD25 PE, CD28 PE, and CD56 PE (Beckman Coulter, Brea, CA, USA) and PD-1 PE, LAG-3 PE, 4-1BB PE, and TIM-3 PeCy-7 (BioLegend, San Diego, CA, USA). Antibodies were pre-titrated using activated as well as non-activated PBMCs to determine optimal staining dilutions. We detected the cell subpopulation of PBMCs prior to culture and within cultured CTLs by flow cytometric analysis as described previously (22). Briefly, cells were re-suspended in staining buffer and then stained with primary antibody at 4°C for 30 min in the dark. Stained cells were centrifuged for 10 min at 1,500 rpm at room temperature and subsequently washed in staining buffer twice prior to fluorescence-activated cell sorting (FACS) analysis. Three-color flow cytometric analysis was run to determine cell phenotypic using Cytomics FC500 and CXP analysis software (Beckman Coulter, USA). CD8^+^PD-1^+^ T cell sorting was carried out using the MoFlo Astrios EQ (Beckman Coulter, USA). First, CD8^+^ cells were enriched using CD8 microbeads (BioLegend), and the enriched T cells were incubated with FITC-conjugated-CD8, PC5.5-conjugated-CD3, and PE-conjugated-PD-1 at 4°C for 30 min. Cell sorting was based on the gating strategy (PI^−^, CD3^+^, CD8^+^, and PD-1^+^). The sorted populations were expanded to detect their reactivity on days 13–15 with irradiated allogeneic feeder cells (5,000 rad) pooled from three donors in a T-cell medium supplemented with 10% human AB serum, anti-CD3, and IL-2 (2,000 IU/ml) (Boehringer Mannheim, Germany).

### Assessment of Tumor Recognition and Cytotoxicity Assay

IFN-γ enzyme-linked immunospot (ELISPOT) assay was used to measure the recognition of targets. After 15 days of *in vitro* culture in cell medium supplemented with 2,000 IU/ml of IL-2, at 37°C in 5% CO_2_, cultured T cells were washed and co-cultured, either alone or with HLA-A2+ target tumor cell. In the ELISPOT assays, effector cells (1 × 10^5^) were added to target cell lines (1 × 10^4^) at an E:T ratio of 10:1 per well in a 96-well plate and incubated for 24 h, according to the manufacturer’s instructions. The raw data were analyzed and plotted using CTL Immunospot software (Cellular Technology Limited, Shaker Heights, OH, USA). The identification of greater than 40 spots and twice background was required to report positive T-cell reactivity.

A Cell Counting Kit-8 (CCK-8) was used to detect cytolytic activity. Target cells were plated with effector cells at various effector/target ratios (6.25:1, 12.5:1, 25:1, and 50:1) in 96-well U-bottomed plate for 24 h at 37°C. The supernatants were harvested for absorbance measurement in a microplate reader at 450 nm.

### T-Cell Receptor Sequencing

DNA was extracted from *ex vivo* expanded T cells using a Qiagen DNA FFPE kit, DNA blood kit, or DNA blood mini kit (Qiagen, Valencia, CA, USA). TCR Vβ CDR3 sequencing was performed using the survey (cultured cells) or deep-resolution (PBMCs) Immunoseq platforms. Bioinformatic and biostatistical analyses of productive clones were performed to assess the dynamics of expanded T cells. The TCR Vβ CDR3 sequence diversity at day 15 during the expansion was compared to the initial TCR diversity.

### Phenotypic Analysis of Peripheral Blood Immune Cells

The technique protocol was similar to our previous reports. Peripheral venous blood was obtained from each patient at various time points after ACT infusion. Whole blood (100 μl) was incubated in the dark with a primary antibody at 4°C for 15 min. Anti-CD3-FITC/anti-CD56-RPE (Dako, Glostrup, Denmark), anti-CD3-FITC (fluorescein isothiocyanate), and anti-CD4-RPE, anti-CD8-RPE, anti-CD45RO, and anti-CD4-FITC/anti-CD25-PE (BD Biosciences, San Jose, CA, USA) were used. After hemolysis for 10 min, samples were centrifuged for 10 min at 1,500 rpm at room temperature, then washed twice in phosphate-buffered saline (PBS), and subjected to flow cytometric analysis. Three-color flow cytometric analysis was performed to determine cell phenotypes using an FC500 (Beckman Coulter) and CXP analysis software (Beckman Coulter). Lymphocytes were gated by forward scatter versus side scatter. Analysis was set to collect 5,000 gated events.

### Statistical Methods

Continuous variables were expressed as mean ± SD and compared using a two-tailed unpaired Student’s t-test; categorical variables were compared using χ^2^ or Fisher’s analysis. Life-table estimates of survival time were calculated for the evaluation of progression-free survival (PFS) and OS as the primary end-point, according to the Kaplan–Meier methodology (23). Receiver operating characteristic (ROC) curves were used to confirm the cutoff values of post-/pre-CD8^+^PD-1^+^, CD8^+^LAG-3^+^, CD8^+^TIM-3^+^ T cells, post-/pre-Shannon index, Clonality, Evenness, and post-/pre-TCR subclones. All statistical evaluations were carried out using SPSS software (Statistical Package for the Social Sciences, version 15.0, SPSS Inc., Chicago, IL, USA) and GraphPad Prism 5 (version 5.01, GraphPad Software, Inc., San Diego, CA, USA). A value of p < 0.05 was statistically significant in all the analyses.

## Results

### Patient Characteristics

A total of 177 patients with PDAC underwent surgical resection in Zhongnan Hospital of Wuhan University from February 2013 to July 2019. Patients were divided into two groups including PD-L1 expression high (n = 67) and PD-L1 expression low (n = 110). The characteristics of all patients are detailed in **Table 1**. There were no significant differences in BMI, estimated blood loss, hospital length of stay, and the proportion of R0 resection between these two groups. Concurrently, 32 patients with advanced PDAC were enrolled in this study at the Capital Medical University Cancer Center, Beijing Shijitan Hospital from June 1, 2013, to May 30, 2019. The characteristics of all patients are detailed in **Table 2**. The majority had metastatic disease and multiple sites of disease, and 56% were PS 2.

**Table 1:** Demographics and baseline characteristics of patients with PDAC who underwent tumor resection.

Variable	High expression of PD-L1 (67)	Low expression of PD-L1 (110)
**Sex**		
Female	28	50
Male	39	60
**Age (years)**	57.6 (35–75)	56.8 (38–80)
**ASA score classification**		
2	30	58
3	37	52
**BMI, kg/m^2^**	24.2 ± 4.7	25.1 ± 5.4
**Pre-operation Serum CA-199, U/ml**		
<37	20	69
≥37	47	41
**Tumor and pathologic characteristics**		
**AJCC TNM stage**		
I	11	23
II	35	78
III	21	9
**Grade**		
G1	5	12
G2	21	48
G3	32	33
G4	9	17
**Tumor size (cm)**	2.32 ± 1.65	2.53 ± 1.77
**Neural invasion**		
Yes	25	43
No	42	67
**Vascular invasion**		
Yes	45	42
No	22	68
**Nodal status: ypN**		
0	17	57
1	38	39
2	12	14
**Tumor resection**		
R0	52	77
R1	15	33
**Operation type**		
Whipple	60	92
Distal pancreatectomy	7	18

PDAC, pancreatic ductal adenocarcinoma; ASA, American Society of Anesthesiologists; BMI, body mass index; AJCC, American Joint Committee on Cancer.

**Table 2:** Demographics and baseline characteristics of patients with APC.

Variable	Median/number
**Total enrollment**	32
**Age**	60.4 ± 6.1
**Sex**	
Female	15
Male	17
**ECOG-PS**	
1	11
2	21
**TNM staging**	
III	4
IV	28
**Site of metastases**	
Liver	18
Lung	5
Peritoneum	10
Bone	4
Other	8

APC, advanced pancreatic cancer; ECOG-PS, Eastern Cooperative Oncology Group Performance Status.

### Peripheral CD8^+^PD-1^+^ T Cells Were Related With Expression of PD-L1 and the Prognosis of Patients With Pancreatic Ductal Adenocarcinoma Who Received Surgical Resection

**Figure 1:**
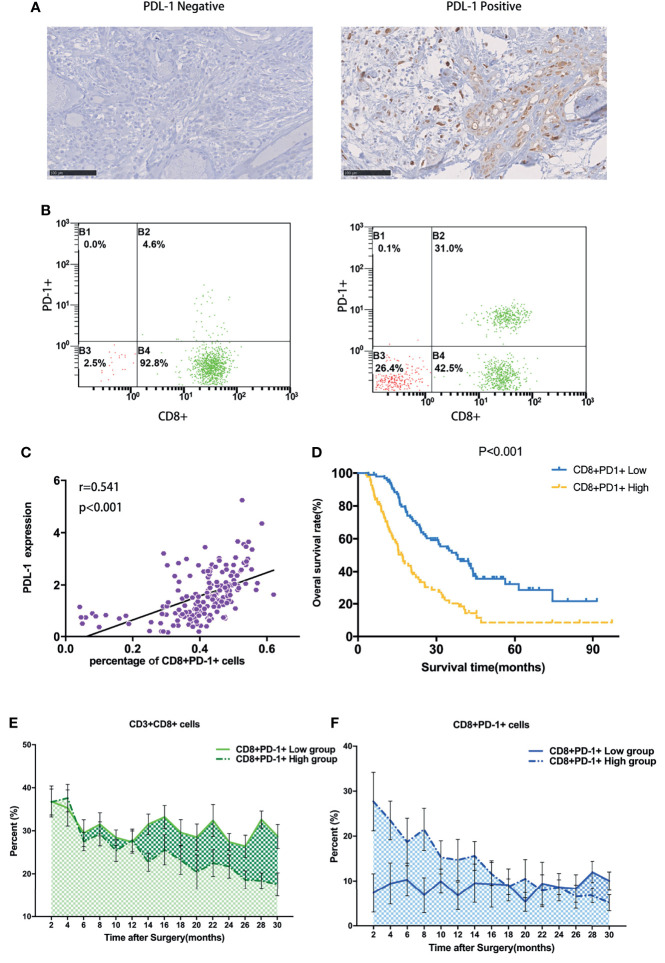
Peripheral CD8^+^PD-1^+^ T cells were related to expression of PD-L1 and the prognosis of patients with PDAC who received surgical resection. **(A)** High expression vs. low expression level of PD-L1 in PDAC. **(B)** The peripheral blood T lymphocyte subtype detection and low peripheral CD8^+^PD-1^+^ T cells vs. high CD8^+^PD-1^+^ T cells detected by flow cytometry. **(C)** The percentage of peripheral CD8^+^PD-1^+^ T cells was significantly correlated with the PD-L1 expression in tumor tissues. **(D)** The high CD8^+^PD-1^+^ T cells were significantly associated with short overall survival. **(E, F)** Tracking test of peripheral blood T lymphocyte subtypes for CD3^+^CD8^+^ T cells and CD8^+^PD-1^+^ T cells for 30 months. PDAC, pancreatic ductal adenocarcinoma.

Of the 177 patients who received tumor resection, 67 tumor samples showed overexpression of PD-L1, and the representative immunohistochemistry (IHC) pictures are shown in **Figure 1A**. Moreover, to determine the prognostic value of PD-L1 expression level in PDAC, we used the Kaplan–Meier method and log-rank test to analyze the relationship between PD-L1 expression and patients’ survival outcomes. We found that high PD-L1 expression in tumor tissues was significantly associated with short OS (**Supplementary Figure 1**). We further detected the peripheral blood T lymphocyte subtypes and found that 80 patients showed a higher percentage of peripheral CD8^+^PD-1^+^ T cells, and the representative pictures by flow cytometry are shown in **Figure 1B**. Interestingly, we found that the percentage of peripheral CD8^+^PD-1^+^ T cells was significantly correlated with the PD-L1 expression in tumor tissues (r = 0.541, p < 0.001, **Figure 1C**). Moreover, the high CD8^+^PD-1^+^ T cells were significantly associated with short OS (**Figure 1D**). We performed a tracking test of peripheral blood T lymphocyte subtypes for 30 months and found that in the high CD8^+^PD-1^+^ T cells, both the CD3^+^CD8^+^ cells and CD8^+^PD-1^+^ cells were decreased, and the changing status is shown in **Figure 1E, F**. Therefore, we hypothesized that the CD8^+^PD-1^+^ cells were exhausted in the high CD8^+^PD-1^+^ T cells group, which was related with PD-L1 expression. Restoring the percentage of CD8^+^PD-1^+^ T cells and combined treatment with anti-PD-1/PD-L1 could be promising treatments for patients with PDAC.

### Phenotypic Analysis of Peripheral Blood T Lymphocyte Subtypes After *Ex Vivo* Expansion

**Figure 2:**
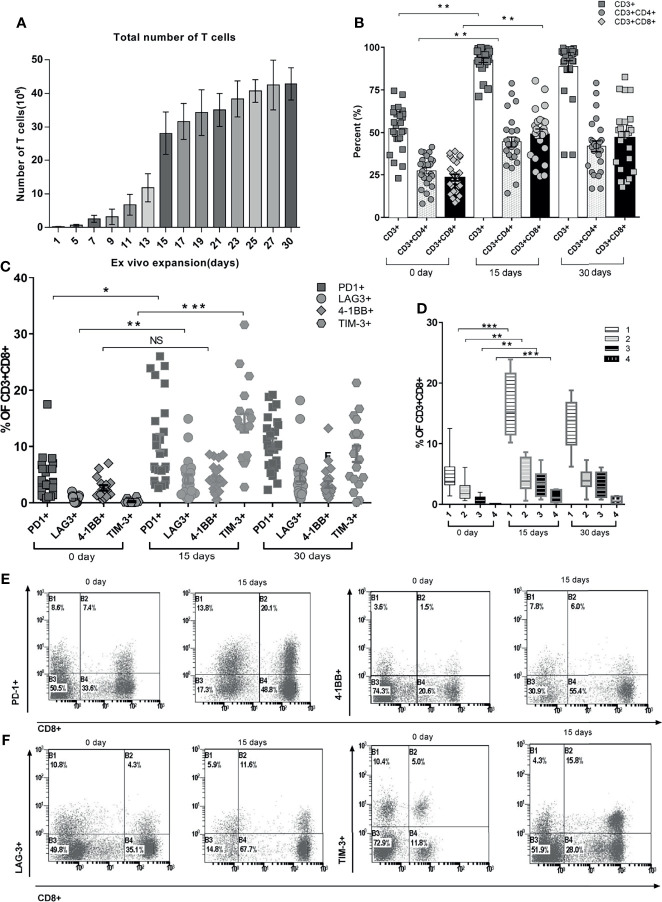
Quantitation of various phenotypic T-cell proportions over time during the *ex vivo* PBMC expansion. **(A)** Total number of expanded T cells daily from day 0 to day 30. Each color represents T-cell assembly from day to day. **(B)** Hierarchic phenotypic percentage of CD3^+^, CD3^+^CD4^+^, and CD3^+^CD8^+^ recorded at day 0, day 15, and day 30. Each actual fraction of T-cell subsets was recorded and shown as mean ± SEM. **(C)** Functional stimulator and suppressor T-cell subsets of each individual were stratified during the continuous expansion at day 1, day 15, and day 30. **(D)** Co-expression percentage of PD-1, LAG-3, TIM-3, and 4-1BB in CD8^+^ PBMCs. The frequency of cells expressing either single or multiple markers above termed as 1, 2, 3, or 4 is shown. Bars represent maximum, minimum, and mean values. **(E)** Fractioned sorting distributions of co-expression percentage of PD-1 and 4-1BB on CD8^+^ PBMCs through cytometric analysis (a representative patient is shown). **(F)** Fractioned sorting distributions of co-expression percentage of LAG-3 and TIM-3 on CD8^+^ PBMCs through cytometric analysis (a representative patient is shown). *p < 0.05, **p < 0.01, ***p < 0.001, Mann–Whitney test. PBMC, peripheral blood mononuclear cell.

Mononuclear cells were harvested from peripheral blood before the treatment of ACT and expanded *ex vivo*. The total number of T cells was 2.57 ± 1.06 * 10^8^ after 7 days, 28.1 ± 6.38 * 10^8^ after 15 days, and 42.8 ± 4.8 * 10^8^ after 30 days (**Figure 2A**, p < 0.001). The CD3^+^, CD3^+^CD4^+^, and CD3^+^CD8^+^ lymphocytes increased significantly by day 15 compared with those of day 0 (**Figure 1B**, p < 0.01), but there was no significant difference between days 15 and 30 (**Figure 2B**, p > 0.05). CD8^+^ T cells exhibited enhanced expression of PD-1, LAG-3, and TIM-3 but not the costimulatory receptor 4-1BB after *ex vivo* expansion (**Figure 2C**). TIM-3 was the receptor most overexpressed by CD8^+^ cells after expansion for 15 days compared with the baseline expression, followed by PD-1 and LAG-3 (15.2% ± 3.6%, 10.4% ± 3.2%, and 5.3% ± 2.3%, respectively) (p < 0.01) (**Figure 2C**). The frequency of cells expressing these markers did not change from days 15 to 30. The cumulative frequency of T cells co-expressing at least 2 of these molecular markers was 9.6% ± 3.8% of CD8^+^ T cells on day 15, compared with 3.1% ± 1.6% of CD8^+^ T cells on day 0 (**Figure 2D**). **Figures 2E, F** show the pattern of expression of these 4 receptors after *ex vivo* expansion for 15 days for a representative APC patient. This patient’s CD8^+^ T cells displayed overexpression of PD-1, TIM-3, LAG-3, and 4-1BB.

### Increasing CD8^+^PD-1^+^ T Cells and Combined Treatment With Anti-PD-1 Were Related With Favorable Outcome in Patients With Advanced Pancreatic Cancer

**Figure 3:**
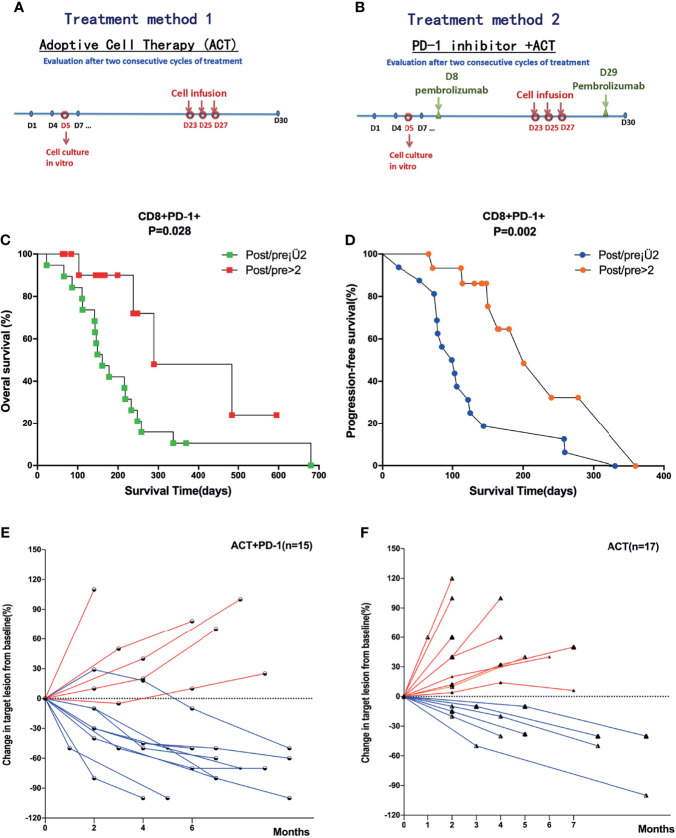
Survival analysis of subgroups divided by the treatment methods and level of CD8^+^PD-1^+^ T cells. **(A)** Treatment method of ACT in all patients with APC. **(B)** Treatment methods for patients received ACT combined with anti-PD-1. **(C, D)** Patients with post/pre > 2 of CD8^+^PD-1^+^ T cells had significantly favorable OS and PFS compared with post/pre ≤ 2 of CD8^+^PD-1^+^ T cells. **(E, F)** The change of the targeted lesion detected by CT or MRI in patients who received ACT combined with anti-PD-1 and solely ACT, respectively. ACT, adoptive T-cell therapy; APC, advanced pancreatic cancer; OS, overall survival; PFS, progression-free survival.

Of all the patients who received ACT, 15 patients received combined treatment of anti-PD-1. The treatment methods are shown in **Figures 3A, B**. The ratios of posttreatment of ACT/pre-ACT CD8^+^PD-1^+^ T cells were detected and calculated. We performed ROC analysis to determine appropriate cutoff values. Survival analysis showed that patients with post/pre > 2 of CD8^+^PD-1^+^ T cells had a significantly favorable OS (median OS time 238 versus 142 days, p = 0.024, **Figure 3C**) and PFS (median PFS time 180 versus 85 days, p = 0.002, **Figure 3D**). Moreover, the change of the targeted lesion detected by CT or MRI showed that the tumor size decreased more significantly in patients who received ACT combined with anti-PD-1 compared with the sole treatment of ACT (**Figures 3E, F**).

### CD8^+^PD-1^+^ T Cells Were Tumor-Reactive Cells and Could Be Applied for Treatment of Adoptive T-Cell Therapy

**Figure 4:**
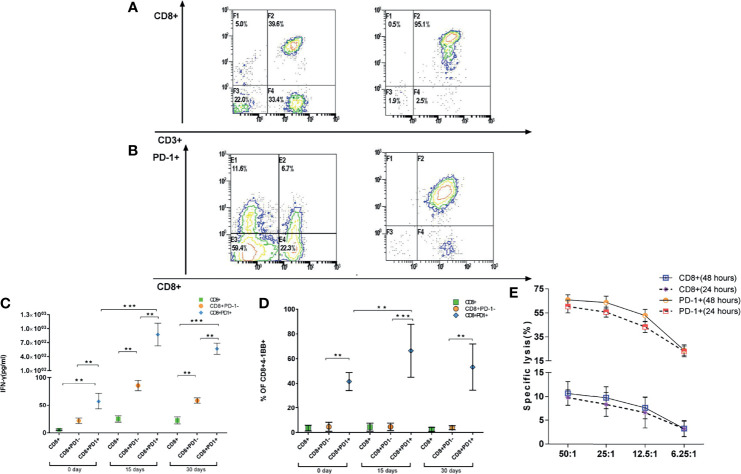
CD8^+^PD-1^+^ T cells could be identified as tumor-reactive CD8 T cells. **(A, B)** CD8^+^ and CD8^+^PD-1^+^ T cells were sorted to identify the tumor-reactive T cells by ELISPOT and CCK-8. **(C, D)** Reactivity of PD-1^+^ and PD-1^−^CD8^+^ T cells derived from patients against autologous tumor cell lines. IFN-γ release and upregulation of 4-1BB (mean ± SD) are shown. **(E)** Lysis of HLA-A2+ matched tumor cells by patient-derived CD8^+^ T cell. *p < 0.05, **p < 0.01, ***p < 0.001, Mann–Whitney test. ELISPOT, enzyme-linked immunospot; CCK-8, Cell Counting Kit-8.

We isolated CD8^+^ (**Figure 4A**) and CD8^+^PD-1^+^ T cells (**Figure 4B**) from patient PBMCs; expanded them *in vitro* for 15 days with IL-2, anti-CD3 stimulation, and irradiated feeders; and tested their ability to recognize autologous tumor cell lines by IFN-γ-ELISPOT using HLA-A2+ tumor cell lines as target cells. Notably, CD8^+^PD-1^+^ T cells, but not CD8^+^PD-1^−^ T cells, contained the tumor-reactive cells as determined by IFN-γ secretion and 4-1BB upregulation after co-culture with the autologous tumor cell line (**Figures 4C, D**). Moreover, after expansion for 15 days, the IFN-γ secretion and 4-1BB upregulation were enhanced (**Figures 4C, D**). Additionally, CD8^+^PD-1^+^ T cells could lyse the HLA-A2+ matched tumor cell lines and have stronger killing efficacy (**Figure 4E**). These data indicate that PD-1 expression identifies tumor-reactive peripheral blood CD8^+^ T cells, indicating that expression of PD-1 after *ex vivo* expansion may be used to prospectively identify and select a repertoire of CD8^+^ tumor-reactive cells.

### T-Cell Receptor Diversity After Expansion Is Associated With Clinical Outcomes

**Figure 5:**
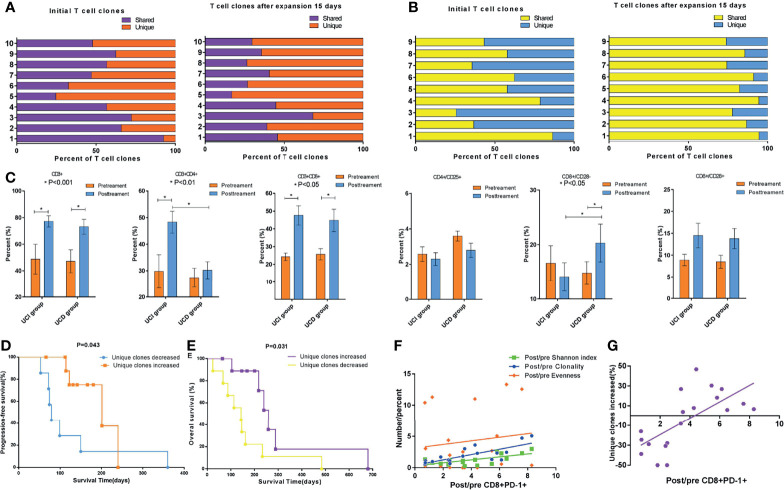
Changes in TCR subclones during *ex vivo* T-cell expansion and the association of CD8^+^PD-1^+^ T cells with TCR diversity and subclones. **(A)** The unique TCR clones increased and the shared TCR clones decreased after T-cell expansion *ex vivo* in 10 patients (p < 0.05). **(B)** The unique TCR clones decreased and the shared TCR clones increased after T-cell expansion *ex vivo* in 9 patients. **(C)** Peripheral blood T-cell phenotype measurements *via* cytometry before and after the first cycle of ACT cell therapy divided by alteration of unique TCR clones. **(D, E)** Survival analysis showed that the group with increased unique TCR clones had significantly longer PFS (p = 0.043) and OS (p = 0.031). **(F)** Correlation analysis showed that the post-/pre-CD8^+^PD-1^+^ T cells were significantly associated with the post-/pre-Shannon index (r^2^ = 0.484, p = 0.009) and Clonality (r^2^ = 0.579, p = 0.002), but not Evenness (r^2^ = 0.018, p = 0.575). **(G)** The post-/pre-CD8^+^PD-1^+^ T cells were significantly associated with the unique TCR repertoire clones in patients who received ACT (r^2^ = 0.464, p = 0.001). TCR, T-cell receptor; ACT, adoptive T-cell therapy; PFS, progression-free survival; OS, overall survival.

In order to calculate the TCR diversity of cultured T cells *ex vivo*, the Shannon diversity index (24), TCR Clonality (25), and Evenness (26) were used to characterize the diversity of TCR Vβ CDR3 sequences of cultured T cell samples of 19 of 32 patients from whom there was an adequate amount of specimen to perform CDR3 TCR Vβ next-generation sequencing. In light of the foregoing data demonstrating that CD8^+^PD-1^+^ T cells are tumor-reactive, and their expansion correlated with the outcomes of patients treated with ACT and considering the role of the TCR repertoire in the antitumor response, we sought to assess the relationship between the CD8^+^PD-1^+^ T-cell frequency and the TCR repertoire. We first investigated the overlap in T-cell clones in the 19 patients. As shown in **Figure 5A**, the unique TCR clones increased and the shared TCR clones decreased after T-cell expansion *ex vivo* in 10 patients (p < 0.05). However, as shown in **Figure 5B**, the unique TCR clones decreased and the shared TCR clones increased after T-cell expansion *ex vivo* in 9 patients. Phenotypic analysis of PBMCs before the ACT treatment and at the end of the first cycle of therapy was performed. We observed that CD3^+^, CD3^+^/CD4^+^, and CD3^+^/CD8^+^ cell subsets were significantly increased in the group in which TCR unique clones increased (UCI) (**Figure 5C**, p < 0.05) after treatment, while CD3^+^, CD3^+^/CD8^+^, and CD8^+^/CD28^−^ were significantly increased in the group of TCR unique clones decreased (UCD) (**Figure 5C**, p < 0.05) after treatment. Importantly, CD3^+^/CD4^+^ was significantly higher in the TCR UCI group than the UCD group (**Figure 5C**, p < 0.05), and CD8^+^/CD28^−^ was significantly lower in the UCI group than the UCD group after treatment of ACT (**Figure 5C**, p < 0.05). Further survival analysis showed that the unique TCR clone increase was related to the prognosis of APC patients. Specifically, the analysis revealed a significantly favorable OS (median OS time 216 versus 112 days, p = 0.031, **Figure 5D**) and PFS (median PFS time 166 versus 79 days, p = 0.043, **Figure 5E**) in patients in whom the unique TCR clones increased compared to those in whom unique TCR clones decreased. Finally, we performed correlation analysis to explore the relationship between CD8^+^PD-1^+^ T-cell level and the TCR repertoire. The post-/pre-CD8^+^PD-1^+^ T-cell ratio was significantly associated with the post-/pre-Shannon index (**Figure 5F**, r^2^ = 0.484, p = 0.009) and Clonality (**Figure 5F**, r^2^ = 0.579, p = 0.002), but not Evenness (**Figure 5F**, r^2^ = 0.018, p = 0.575). Moreover, the post-/pre-CD8^+^PD-1^+^ T-cell ratio was significantly associated with an increase in unique TCR clones in patients who received treatment with ACT (**Figure 5G**, r^2^ = 0.464, p = 0.001).

### Cox Proportional Hazards Analysis to Identify the Significant Prognostic Factors

Cox proportional hazards models were then used to quantify the prognostic significance of risk factors after multivariable adjustment. A multivariable analysis was performed to assess the factors that demonstrated significant effects in univariate analysis. After competing risk factors were adjusted, post-/pre-CD8^+^PD-1^+^ T cells > 2 was an independent prognostic factor for OS (p = 0.009) and PFS (p = 0.012). The details are shown in **Supplementary Figure 2**.

## Discussion

We previously observed improved outcomes in patients with pancreatic cancer who received a combination of S-1 (which has demonstrated antitumor activity in pancreatic cancer) (27) along with ACT immunotherapy, a cell product that includes DCs, T, and NK-T cells. Because this is a heterogeneous population of cells, some of which have antitumor activity but some of which could be potentially immunosuppressive or cause toxicity, an effective biomarker that could specifically identify the repertoire of tumor-reactive and neoantigen-specific CD8^+^ T lymphocytes would be highly advantageous for enhancing clinical efficacy and safety (28). In the present study, we found that among the bulk cell product expanded *ex vivo*, CD8^+^PD-1^+^ T cells could be identified as tumor-reactive, and their expansion correlated with the breadth of TCR Clonality and clinical outcome of patients treated with ACT.

PD-1 expression occurs in response to TCR signaling, and when PD-l binds to its ligands (PD-L1 or PD-L2), it inhibits TCR/CD28 signaling and T-cell activation. Blockade of the PD-1 pathway reinvigorates exhausted T cells and can restore antitumor or antiviral immune responses (29, 30). Therefore, PD-1 expression is often thought of as immunosuppressive; however, T cells that upregulate PD-1 are not always functionally impaired or exhausted. In healthy donors, CD8^+^PD-1^+^ T cells from peripheral blood represent memory effector T cells rather than dysfunctional T cells (31). In advanced melanoma patients, PD-1 is upregulated transiently and often sequentially by neoantigen-specific CD8^+^ T cells upon T-cell activation and exposure to common gamma-chain cytokines including IL-2 *in vitro* (20). Further, we found that CD8^+^PD-1^+^ T cells expanded in IL-2 were capable of secreting IFN-γ and lysing tumor *in vitro*. This suggests that PD-1 may serve as a marker for the reproducible enrichment of tumor-reactive cells for patient treatment. Moreover, patients with post-/pre-CD8^+^PD-1^+^ T-cell ratio >2 in the expanded product had significantly favorable OS and PFS compared with post-/pre-CD8^+^PD-1^+^ T-cell ratio ≤2. This supports the notion that immune dysfunction associated with co-expression of inhibitory receptors on CD8^+^ T cells can be reversed (32, 33) and that the robust expansion of CD8^+^PD-1^+^ T cells may predict clinical benefit from ACT.

We also observed that TCR diversity may increase after expansion and is associated with outcomes in APC patients. The unique TCR clones increased and the shared TCR clones decreased after T-cell expansion *in vitro* in 10 patients, and these patients had a favorable prognosis. Our results suggested that the expression of PD-1 on CD8^+^ T cells captured the diverse repertoire of clonally expanded tumor-reactive lymphocytes and tumor-reactive clones might be highly expanded in the CD8^+^ population and preferentially expanded in the PD-1^+^ population.

CD28 is a co-stimulatory molecule that plays multiple roles in the activation, proliferation, and survival of T cells (34, 35). Accumulating evidence indicates that CD8^+^CD28^−^ T cells are associated with inflammation-related disorders. Meanwhile, CD8^+^CD28^−^ T cells are found in tumor microenvironments and the circulation of cancer patients. Both active and suppressive antitumor immune responses have been ascribed to CD8^+^CD28^−^ T-cell populations (36, 37). We found that CD8^+^CD28^−^ T cells were significantly increased after expansion in the group of patients with a decrease in unique TCR clones (UCD) after expansion and CD8^+^CD28^−^ T cells were significantly lower in the group with an increase in unique TCR clones (UCI). Further, TCR repertoire spectrum typing and sequencing were important for identifying whether CD8^+^CD28^−^ T cells could recognize tumor antigens.

In summary, this study showed that the CD8^+^PD-1^+^ T-cell subgroup was related to expression of PD-L1 and the prognosis of patients with PDAC who received surgical resection. The CD8^+^PD-1^+^ T cells were gradually exhausted, and restoring it by treatment of ACT was associated with significantly favorable OS and PFS. Moreover, combined ACT with anti-PD-1 was effective and promising in patients with APC. Further clinical trials are needed to verify these data.

## Data Availability Statement

The original contributions presented in the study are included in the article/**Supplementary Material**. Further inquiries can be directed to the corresponding authors.

## Ethics Statement

The studies involving human participants were reviewed and approved by Zhongnan Hospital. The patients/participants provided their written informed consent to participate in this study.

## Author Contributions

Conceived and designed the experiments: GQ, YY, and QZ. Performed the experiments: LH, CX, DG, SW, JZ, YS, BL, ZC, and ZY. Statistical analysis: GQ and QZ. Wrote the paper: GQ, YY, and QZ. All authors read and approved the final manuscript.

## Funding

This work was supported by the National Natural Science Foundation of China (82002589) and the Program of Excellent Doctoral (Postdoctoral) of Zhongnan Hospital of Wuhan University (Grant No. ZNYB2019007).

## Conflict of Interest

The authors declare that the research was conducted in the absence of any commercial or financial relationships that could be construed as a potential conflict of interest.

## Publisher’s Note

All claims expressed in this article are solely those of the authors and do not necessarily represent those of their affiliated organizations, or those of the publisher, the editors and the reviewers. Any product that may be evaluated in this article, or claim that may be made by its manufacturer, is not guaranteed or endorsed by the publisher.

## Supplementary Material

Supplementary Figure 1 Survival analysis of subgroups divided by the expression of PD-L1 in the tumor tissues.

Supplementary Figure 2 Cox proportional hazards analysis to identify the significant prognostic factors for OS **(A)** and PFS **(B)**.
