# Supplementary figures and images for: Restored CD8+PD-1+ T Cells Facilitate the Response to Anti-PD-1 for Patients With Pancreatic Ductal Adenocarcinoma
Source: Front Oncol. 2022 Apr 11;12:837560. doi: 10.3389/fonc.2022.837560 (PMC9035626; doi:10.3389/fonc.2022.837560)

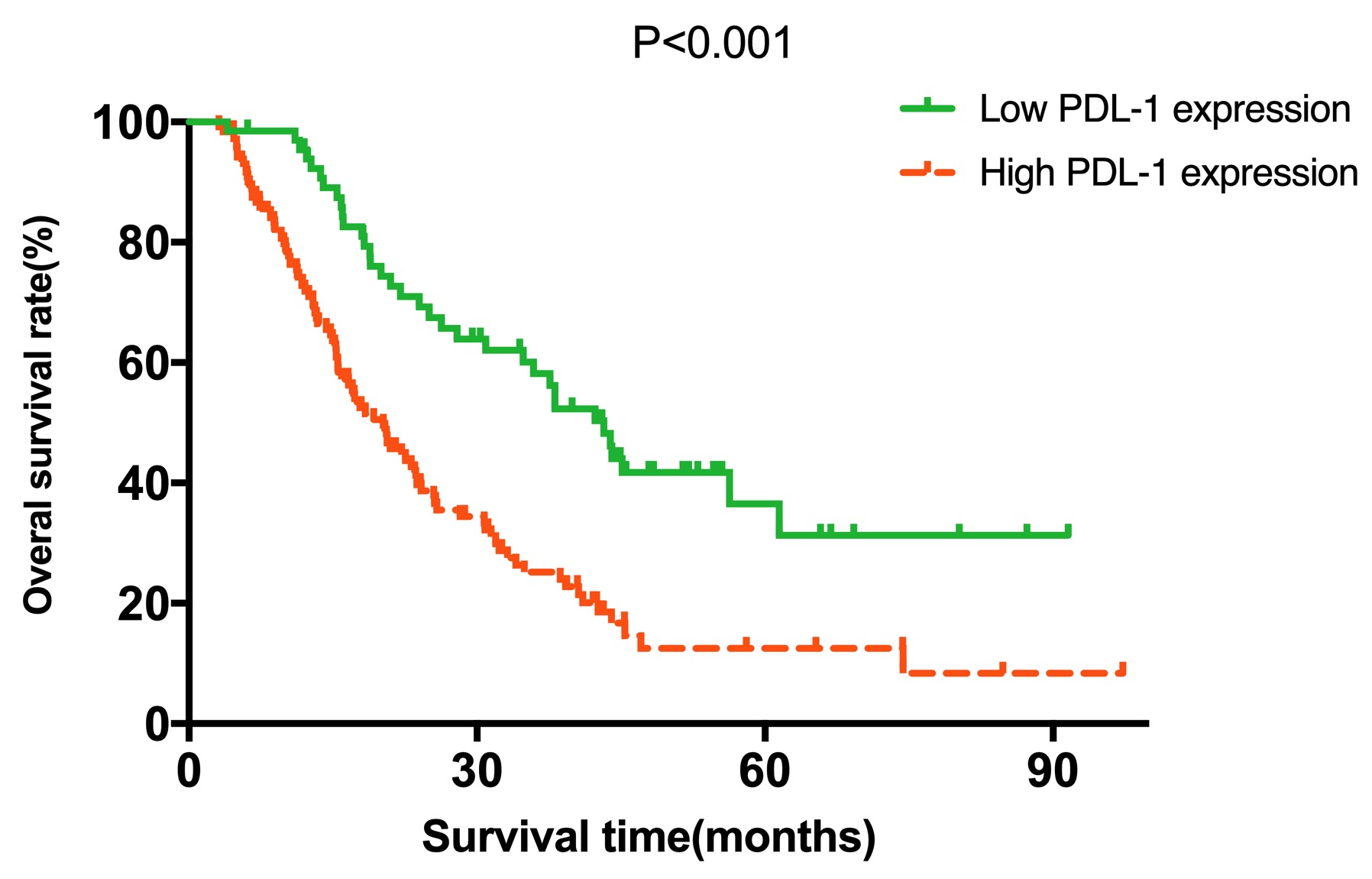

Supplement: Supplementary Figure 1 — Survival analysis of subgroups divided by the expression of PD-L1 in the tumor tissues. [file Image_1.jpeg]

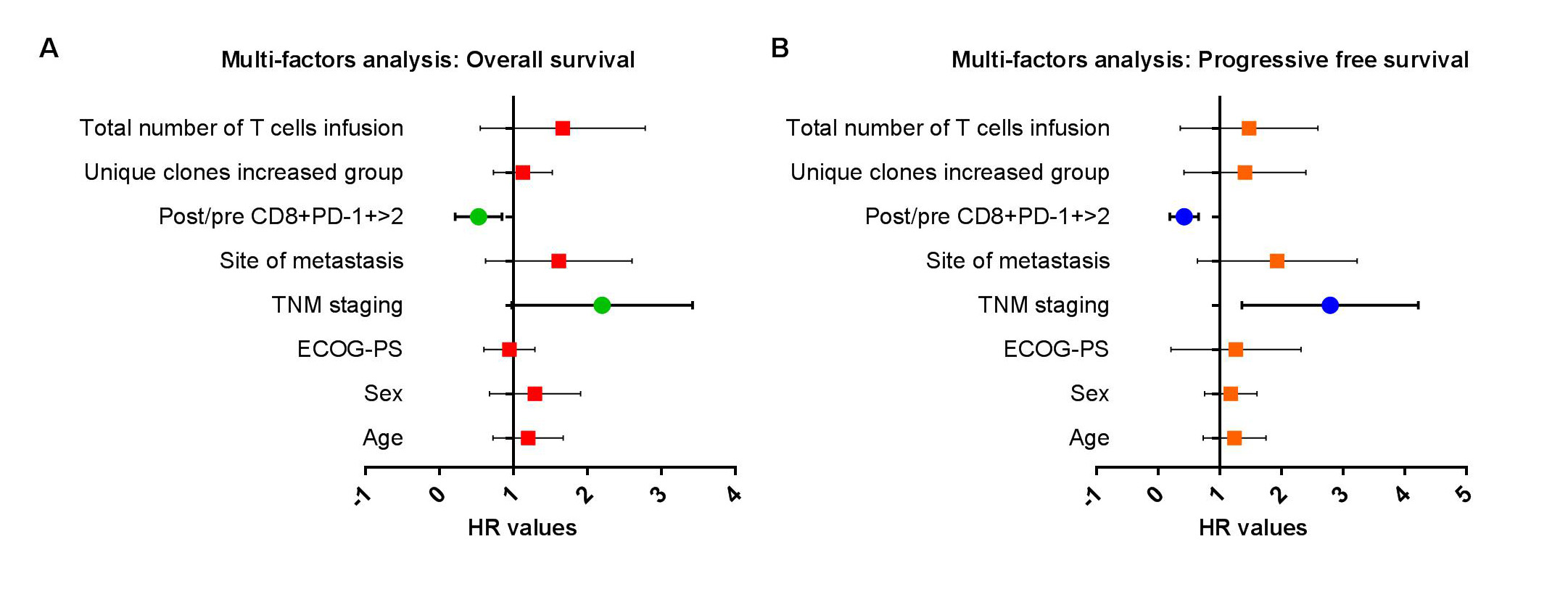

Supplement: Supplementary Figure 2 — Cox proportional hazards analysis to identify the significant prognostic factors for OS (A) and PFS (B). [file Image_2.jpeg]
